# Social information use increases with decreasing winter temperature in a passerine bird

**DOI:** 10.1098/rsos.250180

**Published:** 2025-04-02

**Authors:** Emil Isaksson, Jan J. Wijmenga, Alexis Chaine, Roslyn Dakin, Julien G. A. Martin, Kimberley J. Mathot

**Affiliations:** ^1^ Department of Biology, University of Ottawa, Ottawa, Ontario, Canada K1N 6N5; ^2^ Department of Biological Sciences, University of Alberta, Edmonton, Alberta, Canada T6G 2E9; ^3^ Station d’Ecologie Théorique et Expérimentale du CNRS (UAR2029), 09200 Moulis, France; ^4^ Department of Biology, Carleton University, Ottawa, Ontario, Canada K1S 5B6

**Keywords:** social learning, plasticity, network-based diffusion analysis, harsh environment, cost of learning, starvation risk

## Abstract

Foragers can gain knowledge of profitable foraging opportunities either by sampling the environment directly (asocial information) or from congeners (social information). The relative benefit of using social information over asocial information is context-specific, and social information use is expected to be particularly beneficial when the costs of acquiring asocial information are high, for example, due to high risk of starvation if asocial information fails. We investigated the plasticity of social information use in an overwintering population of black-capped chickadees (*Poecile atricapillus*) as they rediscovered an intermittently available food source. Lower temperatures impose energetic costs that increase the risk of starvation in chickadees; therefore, lower temperatures are predicted to favour higher use of social information. To test this prediction, we evaluated chickadees’ reliance on social information during foraging as ambient temperatures ranged from −11.0°C to 5.5°C. We evaluated the relative strength of reliance on social and asocial information using network-based diffusion analysis. We found increased reliance on social information transmission with decreasing temperature. Reversible plasticity of social information use may be an important mechanism to cope with low ambient temperatures, a seasonal challenge experienced by many animals.

## 1. Introduction

Learning is a cognitive process that allows animals to gather and update information about their surroundings to match their behaviour to current conditions [1–3]. Functionally, learning leads to a change in behaviour based on experience [4]. Asocial trial-and-error learning about resource locations, whereby information is acquired directly from interaction with the environment [5], can be beneficial when resources are abundant because the likelihood of successful personal discovery of resources will be relatively high. In contrast, asocial learning is expected to be particularly costly when resources are limited because individuals are likely to spend more time and energy on unsuccessful sampling of the environment before encountering a resource [6]. Social learning, defined as the use of information about the environment gleaned from the behaviour of others [5,7,8], can alleviate costs related to asocial learning [9–13].

Several evolutionary hypotheses describe how social [14–17] and ecological [18–22] factors have shaped cognition, including relative reliance on social versus asocial learning [11]. Cognitive abilities shaped by evolutionary processes may improve fitness in relation to average environmental conditions over generations. However, these processes are too slow to match within-generation fluctuations in environmental conditions, which instead require plasticity [23,24]. For example, it has been shown that variation in the social [25–27] and physical [28] environments during early-life development can lead to lasting differences in cognitive performance. However, the physical and social environments often change on shorter time scales, including from day to day. Animals should, therefore, also be rapidly and reversibly plastic in their use of different cognitive abilities to best match the current conditions. Indeed, theory predicts that animals should plastically adjust their reliance on social information in response to changes in the cost of asocial information acquisition [11,29,30].

This prediction has been experimentally tested by altering the costs of asocial information gathering, such as predation risk, task difficulty or quality/quantity of food (for overviews, see [13,30,31]). These studies have yielded mixed results. For example, individuals experiencing higher perceived predation risk, in the form of predator scent cues or absence of hiding places within an enclosure, showed higher reliance on social information in several fish species [32–35]. In contrast, Norway rats, *Rattus norvegicus*, generally use social information while foraging under no or low predation risk, but showed no preference for either social or asocial information when predation risk was high [36,37]. Thus, the relationship between asocial learning costs and social information use is both context- and species-dependent. Moreover, while there is some support for plasticity in social learning use when animals are exposed to certain cues in the laboratory, previous studies have not yet examined whether rapid plasticity in social information use also occurs in the wild. Conditions can change quickly in the wild, and we should, therefore, expect the value of social learning to change quickly as well. Measuring plasticity in cognitive expression in relation to a particular cue requires testing the same individuals or groups across multiple states of that cue [38].

The relative importance of social and asocial learning in a population can be evaluated using network-based diffusion analysis (NBDA) [39]. NBDA allows us to estimate the strength of social information transmission in relation to asocial trial-and-error at the group level. The rate of social transmission, or *s*-value, is calculated based on how closely patterns of individual behavioural acquisition match the social connections in the group compared to an estimated baseline asocial learning rate (assuming no social transmission) [39–42]. NBDA is based on the assumption that the likelihood of information transmission between individuals is proportional to their association frequency [39,40], as individuals who interact more frequently should be more likely to learn from one another [43]. NBDA has previously been used to study social learning effects on foraging behaviour in wild populations [44–48]. For example, a recent population comparison in mountain chickadees, *Poecile gambeli*, shows that high- and low-elevation populations that differ in several key ecological variables (e.g. frequency of winter storms and snow depth) differ in their use of social learning while foraging [46,47].

Small birds overwintering at high latitudes face several challenges. The combination of little to no primary production and short day lengths in which to forage [49–51] both restrict energy intake. At the same time, low temperatures significantly increase the costs of thermoregulation, leading to increased overall energy expenditure. Animals are expected to make various species-specific behavioural and physiological adjustments to conserve energy when costs of thermoregulation are high [52–54], including how they invest in learning about foraging opportunities [55]. Temperature varies markedly within seasons at high latitudes, and we should expect to see rapid and reversible plasticity in social information use in response to these fluctuations. Rapid and reversible plasticity in the use of asocial or social information about food patches could play an important role in reducing the risk of energy shortfalls by facilitating resource discovery. During milder winter periods, asocial trial-and-error sampling of the environment to reduce competition for resources may be viable as starvation risk is relatively low [56]. In contrast, social information about potential food can become essential during colder periods as social information use can reduce the variance in food discovery and minimize starvation risk when the baseline risk of starvation is relatively high [56–58].

Here, we test whether winter foraging birds adjust their use of social information depending on daily changes in winter temperature. To do this, we recorded individual visits to feeders providing supplemental food intermittently in flocks of black-capped chickadees, *Poecile atricapillus*. From this, we extracted the order and timing of the first visit to a feeder for every individual in a group after the feeders were refilled. Using NBDA, we then estimated the impact of social transmission on the spread of the information that the feeders were full again within each group. Because temperature dictates energy requirements [59,60], we predicted that social transmission should be negatively correlated with temperature such that at colder temperatures, chickadees would exhibit a higher relative use of social information. This is because social information use is expected to save valuable energy at low temperatures, whereas asocial learning should be less costly (in terms of starvation risk) under warmer, milder conditions.

## 2. Material and methods

### 2.1. Data collection

**Figure 1.**
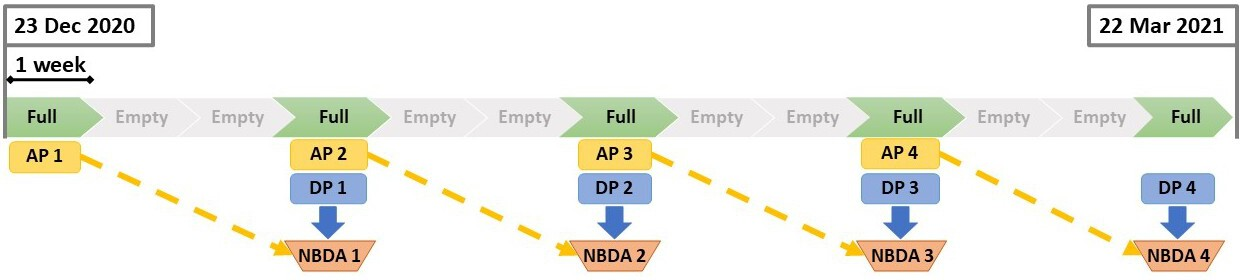
Feeder supplementation schedule illustrating the combinations of social network and diffusion data used to estimate social transmission. Green sections and faded grey sections indicate when feeders were full and empty, respectively. Each section is one week. Note that the diffusion periods and association periods are each illustrated as being one week in duration, because we considered data for this entire duration for the variables calculated during each of these periods. However, in practice, information diffusion was completed in less than 1 week. Yellow boxes indicate the association periods (AP1−4) used to construct social networks. Blue boxes indicate the diffusion periods (DP1−4) investigated for social transmission with network-based diffusion analysis (NBDA1−4, orange buckets). Dashed arrows indicate how each association period was used for the subsequent NBDA.

Birds were captured using mist nets between October and December 2020 at the University of Alberta Botanical Garden in Devon, Alberta, Canada (latitude: 53.407; longitude: −113.758). Each bird was fitted with a unique passive integrated transponder ring (PIT-tag) before being released [61]. Following the capture period, supplemental feeding manipulations were conducted repeatedly from December to March (figure 1). Within the study site, eight custom-made feeders were placed at least 270 m apart [61]. Each feeder had an RFID reader (Priority 1 Design, Australia), which recorded the date, time (nearest second) and PIT-tag ID of each bird that accessed the feeder. Throughout the feeding manipulation period, the feeders alternated between being filled with black-oil sunflower seeds (1 week) and being empty (2 weeks). Each feeder received a similar number of visits across all periods when they were full (electronic supplementary material, figures S1.1–S1.8).

Assessing social information use with NBDA requires information about how a behaviour diffuses throughout a group as well as how individuals in a group are associated. Here, the behaviour of interest was rediscovery of the feeders following an empty period. Each time a feeder was refilled, individuals needed to acquire the information that the feeder once again provided food (diffusion period, DP, 1−4, figure 1). We estimated how individuals were connected to each other based on their visitation behaviour from the preceding full period (association period, AP, 1−4, figure 1). Thus, the social transmission was estimated with NBDAs (figure 1; see §3.2) at each feeder for each DP (henceforth *feeder–period*, i.e the unique combination of feeder and DP) using the feeder visitation behaviour of the focal diffusion period combined with the patterns of association between individuals from the preceding association period. Due to brief battery failures, visits were not recorded from two feeders during periods ‘AP2/DP1’ and ‘DP4’, respectively. As both association and diffusion information is needed to construct the NBDA, we were only able to construct NBDAs for 29 out of the possible 32 total feeder–period combinations.

### 2.2. Weather data

Hourly temperature data were acquired from the Edmonton International Airport (EIA) weather station, approximately 15 km from the study site (climate.weather.gc.ca/historical_data, accessed 8 April 2022). The weather data from this station are representative of the region (see electronic supplementary material in [62]). While the temperatures recorded at EIA may have differed slightly from the temperatures in the garden due to differences in local scale weather conditions, that would only reduce the precision of the temperature data we used relative to the actual conditions in our study site, reducing our statistical power. Thus, any estimated temperature effects would be conservative. For the analysis, we calculated the average daytime temperature across the hours when birds were active at feeders for the first day of each diffusion period (DP1−4, figure 1). We also calculated daily average temperatures across the season to assess the temperature trend across the season (see electronic supplementary material, S2, for details on the weather data).

## 3. Statistical analyses

All data manipulations, calculations and analyses were conducted using R (v. 4.2.2) [63]. Variations in ambient temperature will influence energy requirements, which in turn can influence how frequently birds visit the feeder. As the social network structure and the NBDA used in this study are based on feeder visitation behaviour, the comparability of the estimated social transmission across our study period could be sensitive to temperature variation via temperature’s effect on feeder visits. We therefore used a generalized linear mixed effects model to evaluate the potential influence of temperature on feeder visitation rates (electronic supplementary material, S3). The number of visits was modelled against daily average temperatures as a fixed effect, and we accounted for differences among individuals, and among feeders, by including them as random effects. We used a Poisson error distribution in the model due to the count nature of the response variable. We also added an observation-level variable in the analysis to correct for overdispersion in the data. Mixed effects models were constructed using the ‘*lme4*’ package (v. 1.1-30) [64], with additional significance tests from the ‘*lmerTest*’ package (v. 3.1-3) [65]. Model assumptions were evaluated using the ‘performance’ package (v. 0.12.4) [66].

As each of the variables included in our NBDA analyses (see below) was calculated at the level of feeders, we also evaluated the extent to which feeders could be considered independent sampling units using GLMM (electronic supplementary material, S4). Based on earlier studies documenting winter flock territory sizes for black-capped chickadees [67], we expected that the spacing of feeders in our study site (i.e. minimum 270 m between adjacent feeders) was far enough that each feeder would be on a distinct winter flock territory, and thus represent relatively independent sampling units [68].

### 3.1. Association index

As the NBDA requires information about social relationships within a group to estimate social transmission, we first quantified inter-individual associations at each feeder in each association period. To estimate how individuals at a feeder within a period were associated with one another, we constructed social networks using a modified version of the *Simple Ratio Index*, SRI [69] (AP1−4, figure 1). The SRI requires observations of discrete grouping events, which are commonly identified using an algorithmic approach [45,70–72] such as *Gaussian mixture models* (GMMs) [73]. However, the stream of visits in our study was sometimes too dense for appropriate separation using GMMs (electronic supplementary material, figure S5.1). To accommodate our dense data structure, we used a modified version of the SRI calculation (modSRI). Our modSRI association index was calculated for every possible combination of two individuals (dyads) at each feeder, separately, within each association period. The modSRI was calculated as the number of times two individuals of a dyad were detected at least once within ±20 s of each other, divided by the total number of visits to the feeder made by both the individuals (see electronic supplementary material, S4, for derivation of the modSRI). This gives a single index measure, ranging from 0 to 1 for each dyad, per association period while accounting for among-individual differences in visitation rates. We chose ±20 s as the threshold based on live observation of the birds’ behaviour around the feeder. Birds often perched in the trees around the feeder for several seconds before approaching and often consumed retrieved seeds over a period of several seconds in nearby trees as well. Thus, we deemed ±20 s to be appropriate for capturing most opportunities for social interactions. We repeated all analyses using a ±30 s threshold for the modSRI calculation and confirmed that all of our conclusions were qualitatively similar, which they were (electronic supplementary material, table S6.1).

### 3.2. Network-based diffusion analysis

Social transmission rate was estimated using functions in the ‘NBDA’ package (v. 0.9.6) [74]. We initially used continuous ‘time of acquisition diffusion analysis’ (TADA) [42]. However, comparisons between asocial and social TADA models with different baseline learning functions revealed poor convergence of several models and highly variable *s*-value estimates between baseline learning function. In light of this, we continued with ‘order of acquisition diffusion analysis’ (OADA) as recommended by Hoppitt *et al*. [40] and Hasenjager *et al*. [41]. In this case, the order in which individuals made their first visit was used as our measure of behavioural acquisition.

We constructed OADA models for each feeder–period combination. Individuals that habitually use the feeders while they are full should have higher probability of asocial rediscovery compared to individuals that visit the feeders less frequently. Thus, we included individual feeder use, mean-centred and scaled within feeder and period, as an individual level variable (ILV) affecting only the asocial learning rate in all models. To evaluate the extent to which feeder rediscovery was facilitated by social information transmission, we used a model comparison approach [41,42]. We compared social and asocial OADA models for each feeder–period combination. The relative fit of models was evaluated using the Akaike information criterion corrected (AICc) for sample size. We then used Fisher’s exact test to investigate frequencies with which social and asocial models provided the best model fit across the season.

Estimates of the *s*-value to be used in further analyses were obtained from the models with the lowest AICc value for each feeder–period combination. For feeder–period combinations where the best model was the asocial version, we fixed the social transmission estimate to 0 but extracted the feeder use coefficient from the asocial models. We included the number of visits by an individual standardized by the mean number of visits across individuals as a moderator on the baseline asocial learning rate. Therefore, the *s*-values represent the relative strength of social information use in relation to the estimated baseline asocial acquisition rate by an individual with an average feeder visitation rate within each feeder–period combination.

A limitation of *s*-values is that they are also strongly dependent on the average of the social association indices within a group and thus are not directly comparable across networks [41]. To address this issue, we instead estimated the proportion of acquisition events that can be attributed to social transmission (pST). pST is derived from the *s*-values, provides a standardized estimate of social transmission, relative to asocial acquisition, varying between 0 (completely asocial) and 1 (completely social) and should be comparable across analyses and contexts [41]. We estimated 95% confidence intervals (CI) of the *s*-values using a profile likelihood approach from which we then extracted the 95% CI of pST, following the method outlined by Hasenjager *et al*. [41]. For instances when the profile likelihood method was unable to detect an upper limit of 95% CI, we manually assigned an upper limit of 1 for the analysis.

### 3.3. Proportion of social transmission in relation to temperature

To estimate the relationship between pST and temperature, we needed to consider the uncertainty in the estimate of pST and carry it forward in the analysis. We assumed that the logit transformation of pST followed a Gaussian distribution. With this assumption, we could compute the standard error (logit-se) of each pST estimate on the logit scale as the range of the logit-transformed 95% CI divided by 3.92. We then used an inverse variance weighting approach, a common method in meta-analyses [75,76], to take into account the error associated with each pST estimate. To facilitate logit transformation, we added 0.00001 to all pST = 0 and subtracted 0.00001 from all pST = 1. The same adjustment was done for the upper and lower 95% CI when they were estimated as 0 or 1, respectively. Fixing pST to 0 for feeder–periods when the asocial OADA models provided the best fit also meant that their estimated standard errors were 0. To address this, we instead used the average standard error across feeders and periods for these pST estimates. We then fitted an LMM using the logit-pST with the observations weighted by the inverse of the logit-transformed error of the pST (1/logit-se^2^) to give more weight to values estimated with greater precision. We included DP number as a covariate to account for a temporal effect that could be caused by phenological shifts in individual feeder use, sociality, and environmental variables other than temperature across the season. In addition, we accounted for the non-independence of pST within a feeder across DPs by including feeder ID as a random effect.

## 4. Results

### 4.1. Feeder visits

A total of 360 264 visits by 153 individuals were detected at feeders across the study period. Of the 153 individuals, 55 were females, 58 were males and 40 were of unknown sex (i.e. no blood sample or insufficient blood sample for molecular sex determination). Ages in the population ranged from 0 to 4 years, with *n* = 53 age 0, *n* = 49 age 1, *n* = 34 age 2, *n* = 12 age 3 and *n* = 4 individuals age 4, and *n* = 1 individual with missing age data. However, as we did not have *a priori* hypotheses about age and/or sex differences in information use, and because we had many birds of unknown sex, these variables were not included in analyses and are not discussed further. There was a statistically significant (*z* = −5.191, *p* < 0.001) but biologically negligible negative effect of temperature on individual feeder visits across the study period (average daily temperature slope = −0.007, standard error = 0.001), with only 17 visits difference between −40°C and 6°C ( electronic supplementary material, figure S3.1).

Individuals showed a strong feeder preference throughout the study period. Most individuals (*n* = 80) visited a single feeder. Of the birds that were detected at 2 or more different feeders over the course of the study (*n* = 73; electronic supplementary material, figure S4.1), they demonstrated clear preference for a single feeder (see electronic supplementary material, S4, for details on feeder fidelity). The group size across feeders throughout the study ranged from 8 to 42 individuals (electronic supplementary material, S9). The proportion of matching individuals between the association periods and the DPs ranged from 0.33 to 1, with 18/29 feeder–periods having a proportional match above 0.7 (mean = 0.75, median = 0.81; see electronic supplementary material, table S9.2).

### 4.2. Order of acquisition diffusion analysis and model comparison

**Figure 2.**
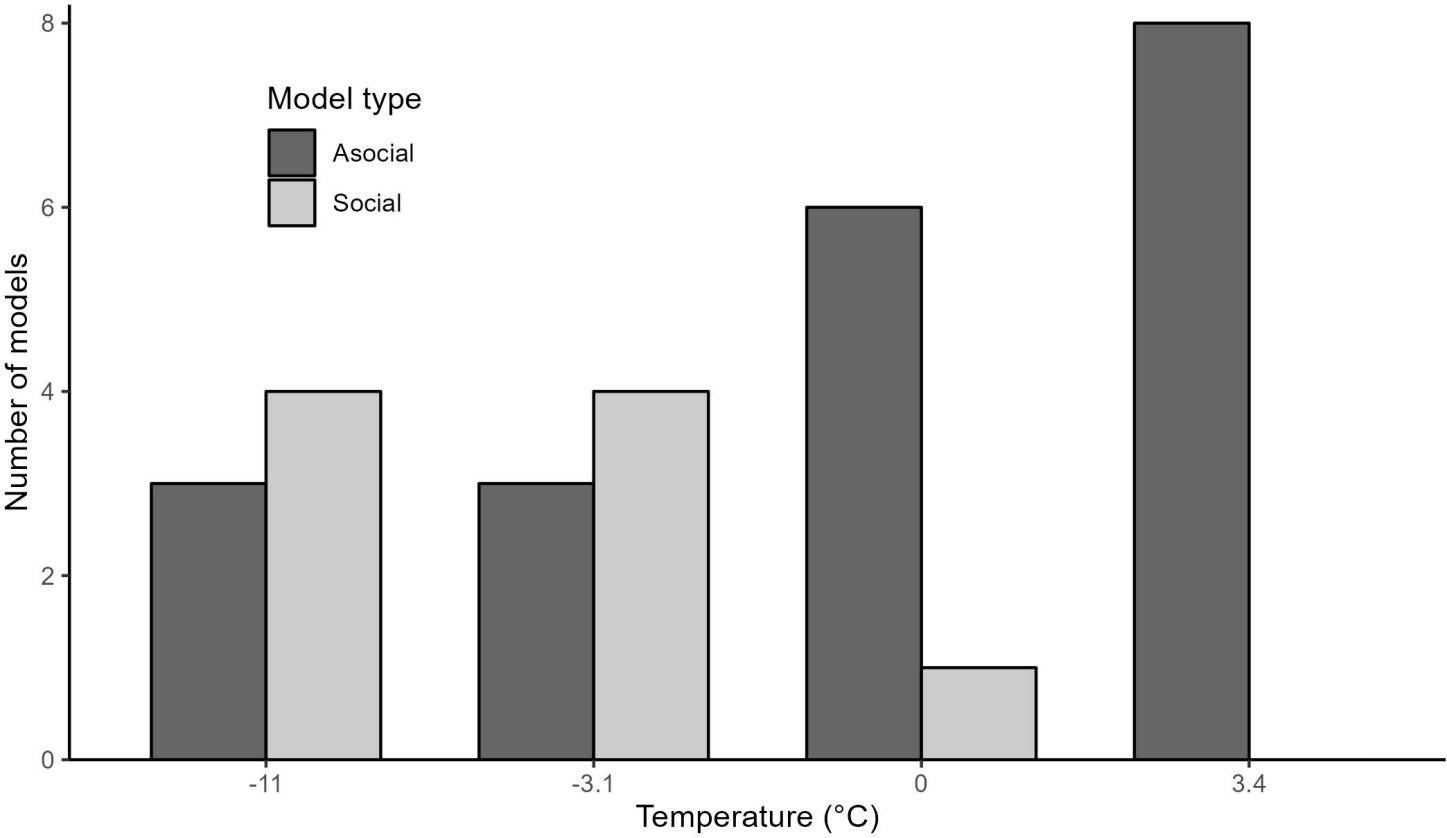
Number of best fitting social (light grey) and asocial (dark grey) OADA models over mean temperature per diffusion period.

Out of the 29 feeder–period OADAs, the social models provided the best fit in 11 instances and the asocial models in 18 instances. Two of the 11 top social models indicated convergence issues and were replaced by asocial models. Further analysis is thus based on values obtained from 9 social and 20 asocial models. The frequency with which social models provided the best model fit was significantly different across temperatures (Fisher’s exact test; *p* = 0.02). Social models provided the best fit more frequently at lower temperatures (figure 2), which coincided with the first two DPs. The social models provided the best fit at 4/7 feeders in the first DP, 4/7 in the second DP, and 1/8 in the fourth DP (figure 2).

In 3 of the social models, the OADA procedure estimated the *s*-value as a disproportionately large positive value. This may be (i) caused by a high positive value of an ILV or (ii) because the behavioural diffusion closely follows the underlying network [41]. The estimates of feeder use for these cases were not disproportionately higher than the estimates of feeder use for the other social models; thus, we assumed scenario (ii), which makes the upper limit of the 95% CI of the *s*-value go towards infinity. In these cases, we fixed the upper limit of pST to 0.99999 as described above.

In 17 of the 29 models, the 95% CI for the feeder use estimates overlapped zero, which indicates that individual feeder use did not have a substantial influence on the asocial baseline acquisition rate (electronic supplementary material, table S9.1). The remaining 12 models estimated feeder use to have a positive influence on the asocial baseline acquisition rate (see electronic supplementary material, table S9.1).

### 4.3. Social transmission in relation to temperature

**Figure 3.**
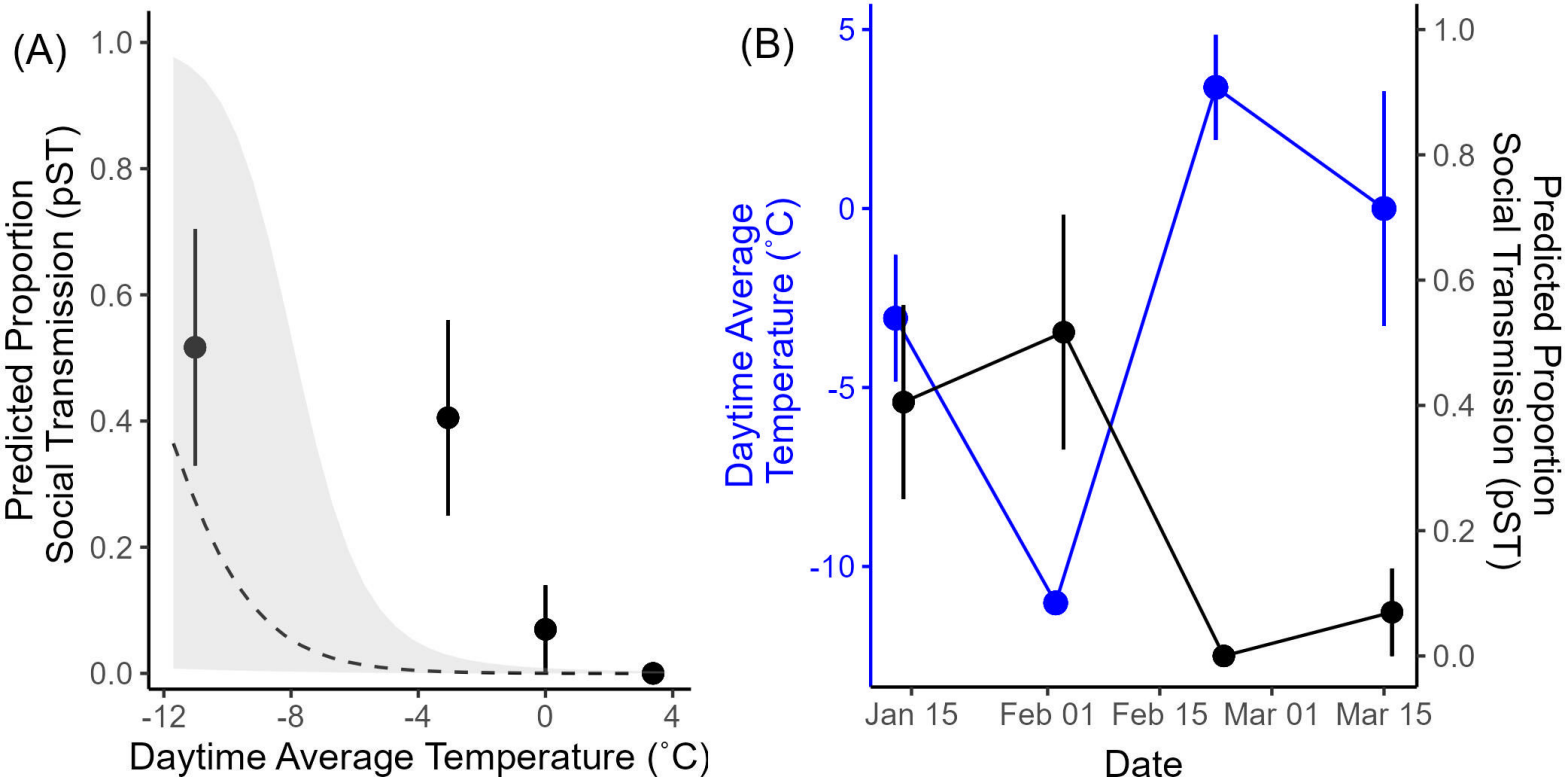
The relationship between pST and average daytime temperature directly (A) and over time (B). Both outer *y*-axes show the pST, with black points and error bars representing the mean ± standard error of the estimated pST across feeders during each DP. The dashed line and grey areas in (A) represent the model predictions from the model in table 1. The left *y*-axis in (B) indicates the temperature range, with blue points and error bars representing the mean ± standard error of daytime average temperature. Lines in (B) are simply linking the points for easier visualization.

For the 9 supported social models, the pST varied greatly across the season (pST range 0.47–1; electronic supplementary material, S10). As predicted, we found a significant negative relationship between pST and average daytime temperature, indicating that when temperatures were colder, the transmission of information about food presence followed the underlying social networks more closely (figure 3A). The LMM results indicate a negative relationship between pST and temperature (table 1). Additionally, there was no significant relationship between pST and DP number, indicating that social transmission use was driven by temperature rather than by a temporal effect (table 1, figure 3B). Finally, the 95% CI of feeder use overlapped 0 in all 9 social models, indicating that baseline feeder use did not explain variation in asocial learning rate.

**Table 1.** Model outputs for the relation between pST and daytime average temperature (*n* = 29).

	pST logit transformed with inverse variance weighting			
predictors	estimate	s.e.	*t*-value	*p*
intercept	−3.38	4.09	−0.83	0.415
daytime avg. temp	−0.63	0.23	−2.72	**0.011**
diffusion period (DP)	−1.81	1.37	−1.32	0.200
random effects				
residual	4.91			
feeder	0.00			

Statistical significance (*p* < 0.05) is marked in bold.

## 5. Discussion

In this study, we investigated whether plasticity in social information use was related to environmental variation affecting the relative costs and benefits of social versus asocial information use. We predicted that the reliance on social transmission should increase as temperatures decreased based on the assumption that the cost of asocial learning would be relatively high at lower temperatures due to increased energetic costs. Our results provide support for this, as the wild groups of black-capped chickadees in our study exhibited rapid and reversible plasticity in social information use in relation to changes in winter temperature. Specifically, social information use was lowest under relatively mild winter conditions (higher temperatures), and groups increased their use of social information as temperatures decreased. Below, we discuss our results in relation to other studies investigating social information use across measures of environmental harshness and highlight key directions for future work.

Here, we show that black-capped chickadees in our study population increased their relative reliance on social information use as mean daily temperatures decreased. This result is consistent with our assumption that the cost of gathering asocial information about the profitability of the feeders would increase with decreasing temperatures, leading to a higher relative reliance on social information. Given that our study design involved rediscovery of food at known feeder locations, first-hand sampling of a feeder may not be expected to represent a biologically important cost. However, previous work in the same population has shown that most individuals avoid personal sampling of empty feeders [55]. This implies that for many individuals, sampling of empty feeders either is costly or yields no net benefit. However, individuals who did sample the empty feeders experienced a significant benefit in the form of higher annual survival [55], presumably due to faster rediscovery of food. Taken together, these results suggest that asocial sampling is costly in this population and that only individuals in better condition can afford to sample [55].

Given that the energy demands of small birds in winter are known to increase with decreasing temperature [49,59,77,78], the general costs of asocial learning should increase with decreasing temperatures. Thus, our finding that the use of social information about food increases with decreasing ambient temperatures is consistent with the prediction that social learning should be more frequent when asocial learning is costly [11,29,79]. Additionally, the absolute value of social information may increase at lower temperatures. Theory predicts that social learning can work as a variance-sensitive strategy to minimize starvation risk [56–58], which is critical for survival for small birds in winter (reviewed in Brodin [51]). Specifically, under conditions of higher starvation risk (e.g. lower temperatures), higher reliance on social information reduces starvation risk because it reduces variance in food discoveries [56,58]. Thus, the shift to increased social information use at lower temperatures observed in the current study may reflect the increased cost of acquiring asocial information and/or the increased benefits of exploiting social information.

At first glance, our results appear to contrast with the result of previous work that has looked at variations in social information use across gradients in environmental harshness [46,47]. In those studies, Heinen and colleagues [46,47] found that high-elevation populations of mountain chickadees, that experience harsher environmental conditions, have a relatively lower use of social information compared to low-elevation populations. However, we suggest that environmental harshness can be defined by different environmental covariates such as low temperatures (as in our study), or as low predictability (e.g. [46,47]). Although proxies for overarching environmental harshness like altitudinal differences are commonly used (e.g. [46,80–82]), it is important to recognize that environmental harshness *per se* is not predicted to exert a specific effect on the relative use of social versus asocial information; the predicted effect will instead depend on the specific dimension of environmental harshness being considered. For example, while lower temperatures are expected to increase the cost of asocial information acquisition and favour higher use of social information, less predictable environments should favour asocial information use because rapidly changing conditions mean that the value of social information deteriorates faster [11,83,84]. As high-elevation mountain chickadees experience more rapid and severe changes in precipitation and snow cover than the low-elevation population [85], the results of the studies by Heinen and colleagues [46,47] align with the prediction that reliance on social learning should decrease with increased environmental unpredictability. Thus, we suggest that the difference between the result of our study and the studies in the mountain chickadees may stem from a difference in what aspect of environmental harshness is captured in each study. In our study, we believe that snowfall/snow cover is unlikely to have influenced the birds’ decision to rely more on asocial or social information. Chickadees forage mainly in trees and shrubs [86–89] and should, therefore, be mostly unaffected by snow cover. Furthermore, our study area had snow cover on the ground throughout the study period (J.J.W., personal observation), and major precipitation events are relatively rare (climate.weather.gc.ca/historical_data). However, changes in food predictability and costs of thermoregulation are not mutually exclusive. Thus, it would be interesting to compare the relative importance of different dimensions of environmental harshness, such as predictability and energy expenditure, in shaping social information use in the wild, where both dimensions vary independently.

Our study focused on the influence of temperature on social information use. We selected temperature as a key variable of interest because it is known to influence energy expenditure in small wintering birds [49,59,78] and because high-quality measurements of temperature are readily available. We hypothesized that the higher costs of asocial learning resulting from higher energy expenditure at low ambient temperatures would favour higher relative use of social information. While our results are consistent with this hypothesis, an important next step is to investigate changes in the relative use of social information in relation to other environmental variables that also increase energy expenditure. For example, solar radiation and wind speed also influence individual energy expenditure in birds [90,91], and therefore we would predict that lower solar radiation and higher wind speeds would also favour increased use of social information. As the structure of the vegetation around the feeders varied, with some being more exposed than others, solar radiation and wind speed may have had a moderating influence on the effect of temperature on social information use in our study. Furthermore, as solar radiation and wind may vary more across shorter distances than temperature, they may have contributed to the heterogeneity in effects across feeders observed in our study. Considering additional environmental aspects that affect energy expenditure including solar radiation and wind speed will allow future studies to draw stronger inferences about the causal effect of energy expenditure on the relative use of social information.

Although we interpret the observed changes in social information use across DPs as a response to changes in ambient temperatures, we also considered two potential alternative explanations. First, changes in social information use may have been driven by temporal (i.e. seasonal) effects, rather than temperature. For example, if chickadees increased reliance on social information in preparation for the winter season, they should have exhibited the highest reliance on social information in the first DP in January, as this is well into the winter season with a decrease towards the end of the season in expectation of spring. Although social models provided highest support for the order of feeder discovery in the first two DPs of the season, there was no significant relationship between period number and pST (table 1, figure 3B). Thus, we found no support for a linear temporal effect on social information use. Second, previous studies have found that chickadees increase foraging rates with decreasing temperature [55,61]. Variation in feeder use may influence the opportunities for temporally clustered events that meet our criteria for modSRI. Thus, one potential concern is that the variation in traffic at the feeders could generate variation in social network edge weights and, hence, the NBDA estimates used in our study. However, we did not observe strong temperature-related differences in foraging rates in the present study (electronic supplementary material, figure S3.1). Further, our analysis controlled for variation in feeding rates in our estimation of pST by including it as a moderator for asocial learning in the NBDA. Those models showed no significant relationship between individual feeder use and asocial learning rate (electronic supplementary material, table S.7.1). Therefore, the temperature-related effects on social information use cannot be explained by either season or foraging rate, and we conclude that temperature remains the most likely proximate factor shaping variation in social information use across DPs observed in this study.

Although our results are consistent with temperature as a key driver of variation in social information use in chickadees, by chance, one of the four DPs observed had markedly lower ambient temperatures than the other three DPs. This means we cannot address whether the effect of temperature is linear, or whether there is a threshold effect. Furthermore, we were unable to evaluate interactions between temperature, individual feeder use and period of social information use. As feeder visitation remained stable over time (electronic supplementary material, figures S1.1–S1.8) and across temperatures (electronic supplementary material, figure S8.1), we found no indication of potential interaction effects. Nonetheless, further studies with data collected over a wider range of temperatures and with more replicates over time would be needed to evaluate both potential nonlinear effects of temperature, as well as interaction effects.

Overall, our results suggest that changes in winter temperatures are associated with rapid plasticity of social information use, and we highlight a number of directions for future work in this area. We predicted an effect of temperature on the relative use of social information *a priori* based on the assumption that asocial information gain would be more costly at lower temperatures due to increased energy expenditure. We suggest that the key next step to understanding how challenging environments shape social information use is to test the relationship between social information use and other environmental cues that affect animals’ energy expenditure [52–54], such as solar radiation or wind speed. Such studies would provide a strong test of the hypothesis that energy expenditure is the direct proximate factor underlying the temperature-related shifts in social information use observed here. Additionally, while we highlight the fact that different dimensions of environmental challenges (e.g. energetic costs, food predictability, etc.) are expected to be important in shaping social information use, how these challenges interact to shape social information use strategies is unknown. Finally, our finding that social information use varies with ambient temperatures is interesting given that winter temperatures are a major agent of selection for many species living in temperate zones, and hints that rapid reversibility in the use of social information could be adaptive. Studies that quantify individual variation in plasticity of social information are needed to better understand the adaptive value of different social learning strategies.

## Data Availability

Data and code related to the project can be accessed on the Open Science Framework digital repository [92]. Supplementary material is available online [93].
